# Imaging supermolecular interactions of the pharmaceutical-cocrystal of apigenin-nicotinamide binding with serum albumin

**DOI:** 10.55730/1300-0527.3560

**Published:** 2023-04-04

**Authors:** Yuting SUN, Ming GUO, Xiaoxue ZHAO, Ronghui WU

**Affiliations:** 1 College of Environmental and Resource Sciences, Zhejiang Agricultural & Forestry University, Hangzhou, Zhejiang, China; 2 College of Chemistry and Materials Engineering, Zhejiang Agricultural & Forestry University, Hangzhou, Zhejiang, China

**Keywords:** AP-Nico pharmaceutical cocrystal, HSA, drug delivery, interaction, molecular modeling

## Abstract

In this paper, a new pharmaceutical cocrystal was synthesized using apigenin (AP) and pharmaceutically acceptable conformer nicotinamide (Nico), and the drug delivery between AP-Nico pharmaceutical cocrystal and human serum albumin (HSA) in vivo was studied at atomic scale. The pharmaceutical cocrystal was characterized using Fourier-transform infrared (FTIR) spectroscopy, ^1^H NMR spectroscopy, differential scanning calorimetry (DSC), and powder X-ray diffraction (PXRD), and the self-assembling mechanism was explored. The dissolution and cumulative release in vitro were investigated. Molecular dynamic (MD) simulation combined with fluorescence spectroscopy was used to study the delivery mechanism of AP-Nico to HSA. The results showed that AP was pharmaceutically cocrystallized with Nico, which formed a pharmaceutical cocrystal mainly through hydrogen interaction between the -OH groups of AP and –NH_2_ groups of Nico. The solubility of the AP-Nico was 3 times higher than raw AP and the cumulative release rate was 71%. The fluorescence spectroscopy results showed that the AP-Nico pharmaceutical cocrystal bind with Sudlow’s site I inside the HSA molecule with hydrogen-bond interaction as the main force. The Sudlow’s site I of HSA conjugated with AP-Nico explains the conformational changes of HSA in-silico. This study provided a useful reference for synthesizing flavonoid pharmaceutical cocrystal to improve solubility and exploring the interaction mechanism while understanding its delivery mechanism in vivo.

## 1. Introduction

Most active pharmaceutical ingredient (API) extracted from traditional Chinese medicine has a specific biological function. API has a good curative effect in the treatment of corresponding diseases; for example, ursodeoxycholic acid has a good efficacy in disease of primary biliary cirrhosis [1, 2], and paclitaxel is used for treatment of ovarian cancer and nonsmall cell lung cancer [3, 4]. However, clinical observations show that many of the APIs have low bioavailability due to their poor solubility, which affects APIs’ clinical effect. Thus, this issue has become one of the bottlenecks restricting the application and internationalization of Chinese medicine [5]. To solve the problem of poor solubility and low bioavailability of API, many methods have been tried by researchers, including solid dispersion, self-microemulsificiation technology, ultrafine grinding, and liquid-solid compression [6, 7]. However, there are still some problems in these methods. For example, it is difficult for supercritical fluid technology to remove organic solvents. Solid dispersions are prone to aging during storage. Liquid compression is not suitable for the preparation of high-dose insoluble drugs [8, 9], and all of them limit the development of the API’s drug. Therefore, solving the problem of poor solubility and absorption of insoluble API in traditional Chinese medicine is one of the major challenges in the field of pharmacy.

In recent decades, with the development of crystal engineering and supramolecular chemistry, researchers apply those principles to the drug design, forming another major type of drugs—pharmaceutical cocrystal. Pharmaceutical cocrystallization refers to the single system in which self-formed API called pharmaceutical cocrystal former (CCF) that has a fixed stoichiometric ratio through noncovalent interactions [10, 11]. This method of cocrystallization shows great potential, such as solubility, bioavailability, and physical-chemical stability [12, 13]. The most significant application value of cocrystallization in pharmaceutics is to achieve the purpose of modifying physical-chemical properties without changing the covalent structure of drugs. At present, the main focus is on the preparation, formation process, structure characterization, and physicochemical properties of chemical drugs. However, there are few reports on pharmaceutical cocrystal of insoluble API in Chinese medicine. Therefore, in this paper, we used the pharmaceutical cocrystallization method to improve the bioavailability of API.

**Figure 1:**
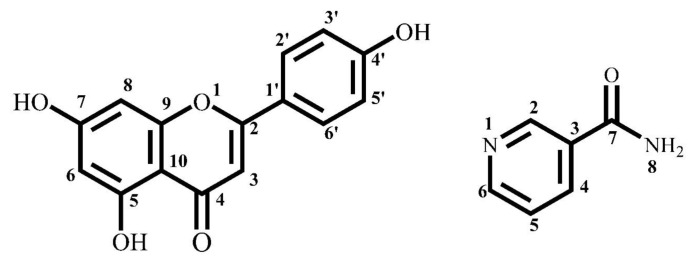
Chemical structures of apigenin and nicotinamide.

Flavonoids are a kind of traditional Chinese medicine with low molecular mass, which are mainly free or combined with glucose as a glycoside. Not only are there wide varieties of flavonoids but also a variety of structural types. However, most flavonoids have poor solubility and low bioavailability, which limits their clinical application [14, 15]. Most structures of flavonoids contain many phenolic hydroxyl groups, which can be used as proton receptor and proton donor, so that it can form hydrogen bonds with CCF. Apigenin (AP) is a typical flavonoid, which has biological activities of antiinflammatory, lowering blood pressure, antiarteriosclerosis, and antithromboembolism [16, 17]. However, the bioavailability of AP is limited due to the low solubility. In this paper, AP and Nico were used to form pharmaceutical cocrystal. It could provide a new sight for the preparation of AP derivates. The chemical structures of apigenin and nicotinamide are shown in Figure 1.

The pharmacokinetics of drugs in vivo is related to absorption, distribution, metabolism, excretion, and toxicity (EADM/T). Drugs reach the receptor sites through the storage and transportation of plasma and then produce pharmacological effects. Moreover, the nonspecific binding of drugs with plasma proteins or components of the body is an important process for drug delivery [18, 19]. Especially, the specific combination of the plasma proteins is the key to the preliminary work of designed drugs. The binding ability of APIs to HSA is a key factor in determining the utilization rate and diffusion from the circulation system to the target [20]. Almost all EADM/T are significantly influenced by drug-protein combinations. However, there are EADM/T studies only in the form of free AP and few reports about the pharmacological mechanisms of its derivatives [21, 22]. Besides, there are two interesting questions, the first one is, how is the interaction between the synthesized AP-Nico and HSA possible? And then what changes will happen to the interaction of AP-Nico to HSA? Hence, it is of great significance to elucidate the transportation process of drugs in vivo. At present, many researches have focused on the interaction between a single substance and a single protein. However, fewer studies have been reported on the mechanisms of pharmaceutical cocrystal to plasma proteins [23]. In this paper, we seek to further study the interaction mechanisms of AP-Nico to HSA and establish interaction models by molecular dynamics simulation. The interaction between AP-Nico and HSA can reflect the EADM/T properties in vivo, so we can have a deeper understanding of the delivery mechanism. It would also be beneficial not only to develop flavonoids which have poor availability but also to provide scientific data for the bioavailability of drugs.

## 2. Materials and methods

### 2.1. Reagents and chemicals

Apigenin (≥98%, HPLC) was purchased from PureOne Biotechnology (Shanghai). Nicotinamide (≥98%) was purchased from Sigma (Aldrich, Shanghai). HSA was purchased from Sloaribio (Beijing, China). Ibuprofen and oleic acid for detecting binding sites were purchased from Sigma (Aldrich, Shanghai). All other chemicals and regenerates are analytical grade. Tris-HCl buffer (0.2 M Tris, 0.1 M NaCl, pH 7.4) was used as a reserve solution for fluorescence spectroscopy experiments. NaAc-HAc buffer (0.02 M. pH 4.5) was used for dissolution rate experiments.

### 2.2. Methods

#### 2.2.1. Preparation of pharmaceutical cocrystal of AP-Nico

Apigenin reacted with nicotinamide, 2-aminometidine, and citric acid in the molar ratio of 1:1, 1:2, and 2:1. Five hundred milligrams of precisely weighed apigenin was placed in a grinding bowl, and small amounts of ethanol were added several times to assist grinding. The mechanochemical method was used to grind at room temperature for 30 min. The collected products were dried in a vacuum drying box, and the apigenin eutectic was obtained. Using AP as API, nicotinamide, 2-aminomidine, and citric acid as CCF, AP eutectic was screened by solvent evaporation method. The product was characterized by PXRD to determine whether there was a new phase. The results showed that the PXRD peaks of AP and citric acid and 2-amidomide complexes were the superposition of the PXRD characteristic peaks of APs and CFCs, indicating that AP and CFCs did not form eutectic, while the PXRD of AP and nicotinamide complexes had new characteristic peaks compared with the characteristic peaks of their physical mixtures, indicating that a eutectic was formed between them. However, the single characterization cannot be accurately explained, and other characterization methods are needed to judge. Therefore, apigenin-nicotinamide is taken as the research objective for later synthesis and research.

AP-Nico pharmaceutical cocrystal was synthesized using the slow evaporation method. The reaction diagram containing the reaction conditions of AP-Nico is shown in Figure S1. AP (1 g, 0.0037 mol) and Nico (0.45 g, 0.0037 mol) in a definite stoichiometric ratio (1:1) were resolved with addition of EtOH solvent (40 mL) for about 12 h at 298 K [24]. Samples were stirred at 2000 rpm in a magnetic stirrer (DF-101 S, Shanghai, China). The product was filtered and evaporated slowly at 298 K. Resulting products were characterized using a series of testing equipment.

#### 2.2.2. Characterization

FTIR Spectroscopy: The FTIR spectrums of AP, Nico, and AP-Nico pharmaceutical cocrystal were obtained in KBr diffuse reflectance mode using a Fourier-transform infrared spectrometer (IRP restige-21, Shimadzu). KBR samples were prepared (1:100). Each sample was scanned 10 times at a resolution of 4 cm^−1^. Samples were scanned at intervals of 2 cm^−1^ from 4000 to 400 cm^−1^. The data was managed by Nicolet Omnic (Version 8.0).

PXRD: PXRD patterns were collected by X-ray diffraction (XRD-6000, Shimadzu, Japan) with Cu-K alpha radiation (1.5406 Å). The tube voltage and current are set at 40 kV and 40 mA respectively. AP and AP-Nico (50 mg, respectively) were scanned between 5° and 40° (2 θ) with step size of 0.02° and time per step of 0.3 s. Appropriate software was used to collect and process the data.

DSC: DSC was conducted on a DSC (TA Co., America) Q 2000 instrument. Accurately weighed AP, Nico, and AP-Nico pharmaceutical cocrystal, respectively, (4–5 mg) were placed in hermetically sealed aluminum crucibles. DSC in the range of 324–624 K was scanned at 284 K/min heating rate in dry nitrogen atmosphere (flow rate 80 mL/min). The data was managed by TA-Universal Analysis 2000.

^1^H-NMR: ^1^H NMR spectra was recorded in DMSO-D_6_ solvents by Bruker Avance II 500 MHz spectrometer, and all chemical position shifts were reported by one millionth (ppm, *δ*).

^13^C-NMR: ^13^C NMR spectra were recorded in DMSO-D_6_ solvents by Bruker Avance III HD 500MHz spectrometer, and all chemical position shifts were reported by one millionth (ppm, δ).

EA: The C, H, and N elements of the sample were determined by Elemental analysis (EA) instrument (Elementar Vario EL cube, Elimonta, Germany).

#### 2.2.3. Apparent solubility determination

The AP and AP-Nico crystals (10 mg, respectively) were determined by suspending them in 25 mL of distilled water and placing them in a 50-mL round bottom flask with glass stoppers. The suspensions were kept at different temperatures of 298 K and 310 K and stirred at 100 rpm using a magnetic stirrer. After 72 h, the sample concentration was measured at 342 nm by ultraviolet-visible spectrophotometer (Shimadzu Corporation of Japan), and the interference of suspension was filtered using a 0.22-μm microfiltration membrane.

Solubility studies: Solubility studies were undertaken on parent AP and AP-Nico pharmaceutical cocrystal in NaAc-HAc buffer and stirring the slurry in a thermostated (310 K) vessel, with a magnetic stir bat at 100 rpm. The suspension was sampled at specific time points (5, 10, 15, 20, 30, 45, 60, 90, 120, 150, 180, 240 min), and filtered using a 0.22-μm microfiltration membrane. The concentration of AP and Ap-Nico crystals was determined by ultraviolet-visible spectrophotometer (Shimadzu Company, Japan) from the slope of absorption and known concentration. Absorbance was measured at 342 nm where no interference of the conformer occurs. And the dissolution rate was obtained according to the cumulative release formula:


(1) $$Q=\text{n}\%=A_{n}+\frac{\left(A_{n-1}+A_{n-2}+A_{n-3}+\cdots \cdots +A_{2}+A_{1}\right)\times V_{1}}{V_{2}}$$

#### 2.2.4. Spectroscopic studies on the interaction of AP and AP-Nico pharmaceutical cocrystal with human serum albumin

HSA reserve solution was prepared in a pH 7.4 Tris-HCl buffer with a final concentration of 1 × 10^−5^ mol/L. The stock solutions of AP and AP-Nico pharmaceutical cocrystal were dissolved in ethanol with final concentration ranging from 0.4 × 10^−5^ mol/L to 4.0 × 10^−5^ mol/L. All fluorescence spectra were measured using an F-7000 spectrophotometer (Tokyo Hitachi Co., Ltd.) in a 1-cm quartz cell. Fluorescence emission spectra at 280 nm were recorded at 298 K and 310 K. The excitation and emission slits were set at 2.5 nm. In the process of fluorescence spectrum analysis, the background fluorescence of buffer solution was removed and the internal filtration effect was eliminated. In the fluorescence spectrum analysis, the background fluorescence of the buffer is removed and the internal filtering effect is eliminated. The internal filtration effect is eliminated according to the following equation:


(2) $$F_{e}=F_{m}e^{(A_{1}+A_{2})/2}$$

In the formula, *F*_e_ and *F*_m_ represent the corrected and measured fluorescence; *A*_1_ and *A*_2_ are the sum of the absorbance of HSA and GS/GS-Adenine at the excitation and the maximum emission wavelength, respectively [25, 26].

#### 2.2.5. Molecular docking and molecular dynamic simulation

The crystal structure of HSA (PDB: 1H9Z) was refined and added with energy. AutoDock 4.2 software (The Scripps Research Institute, TSRI) was used to remove bad contacts. Next, solvent removal and charge calculation of gas generator were carried out. The AP and AP-Nico were obtained from ChemDraw and optimized by Gaussian 09. And 20 conformation searches were carried out by genetic algorithm (GA) [27]. Finally, AP and AP-Nico attained poses were further used for MD simulations exploration.

MD simulations were calculated on AP-Nico/AP-HSA complexes through YASARA (Version) package. We used YASARA Structure to set molecular dynamics of the energy-minimized, physiological pH 7.4, and periodic boundary conditions. Then all complex submitted macro (md_run.mcr) to YASARA for MD analysis at 298 K simulated for 50 ns in an isothermal-isobaric (NPT) ensemble with a time step of 2.5 fs [28, 29].

## 3. Results and discussion

### 3.1. Characterization of AP-Nico pharmaceutical cocrystal

#### 3.1.1. FTIR characterization

**Figure 2:**
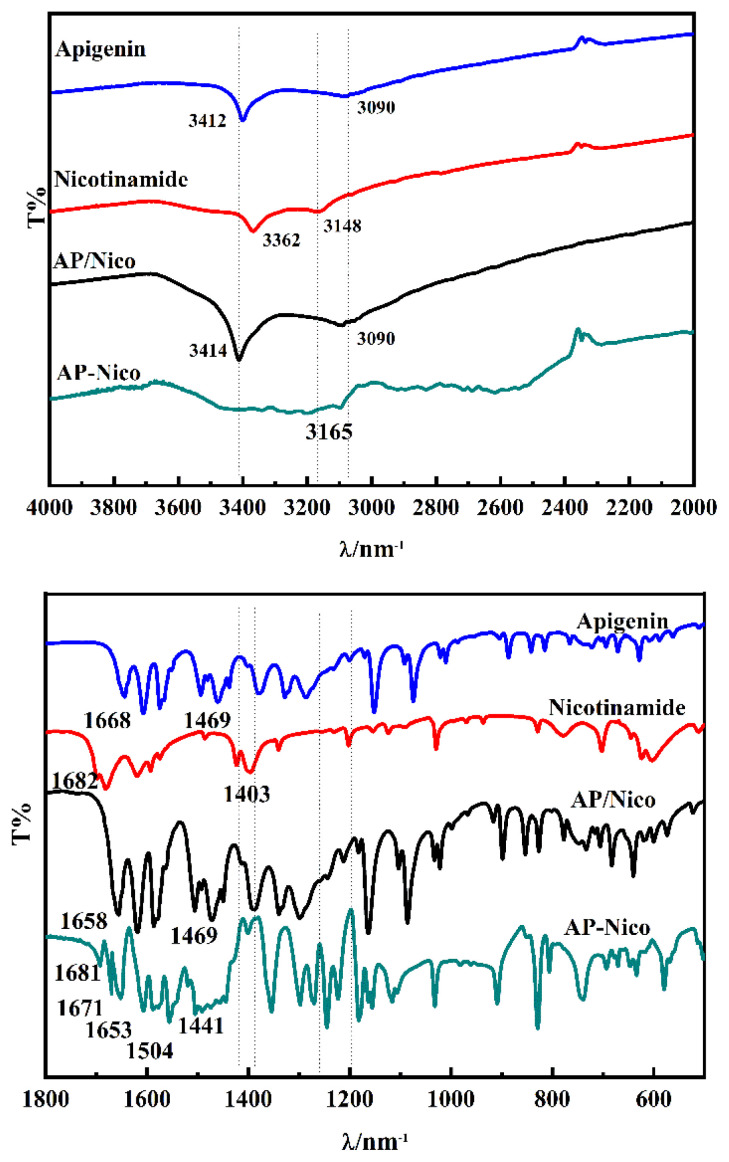
FTIR spectra of apigenin, nicotinamide, AP/Nico mixture and AP-Nico pharmaceutical cocrystal.

FTIR is a relatively reliable method to detect structures of substances. The detailed peak assignment for the free AP, Nico, and AP-Nico pharmaceutical cocrystal is given in Figure 2.

[IR v/cm (KBr)]: AP: 3412 (-OH, v, s), 1658 (-C=O, v, vs), 1602-1356 (-C=C-, v, vs); Nico: 3362 (-N-H, v_s_, s), 3148 (-N-H, v_as_, s), 1682 (-C=O, v, vs), 1403 (C=N-, v, s). The peak position of AP/Nico mixture is the superposition of Apigenin and Nicotinamide, and the FTIR peaks of AP-Nico pharmaceutical cocrystal are obviously different from the spectra of the physical mixture of apigenin and nicotinamide, which indicates that the interaction between AP and Nico does occur during the grinding process. Therefore, AP-Nico cocrystal showed characteristic absorption peaks, including 3165, 1671, 1441 cm^−1^. The peak of the AP-Nico pharmaceutical cocrystal at 1671 cm^−1^ vested in -C=O of AP-Nico, which represented the –C=O peak shifts, and the peak of -C=O was almost unchanged, but the peak of -OH 3165 cm^−1^ was so broad and scattered, it could just be inferred that the peak of -OH was so broad that it coincided with the peak of -NH_2_ group, which may be due to the change of crystal arrangement and structural disorderliness in AP-Nico pharmaceutical cocrystal. C=O of Nico shifts from 1682 cm^−1^ blue to 1671 cm^−1^, and a new peak appears at 1691 cm^−1^. This may be due to the hydrogen atom interaction between AP-OH and Nico-NH_2_ groups and the interaction between Nico-C=O and AP-OH, resulting in AP-Nico pharmaceutical cocrystal. The increased wave-number and broad chemical peaks of the chemical bonds which were involved in the formation of hydrogen bonds due to the intermolecular interactions of AP and Nico [30]. Besides, when Nico was introduced into AP, the original intramolecular hydrogen bonds were destroyed due to the inductive effects of interaction forces [31], so that displacement of -OH characteristic peak appeared. According to the relevant IR spectra, it could be speculated that the products contained AP and Nico, and the hydrogen bonding between -OH in AP and -NH_2_ of Nico was produced in AP-Nico pharmaceutical cocrystal.

#### 3.1.2. 1H-NMR characterization

**Figure 3:**
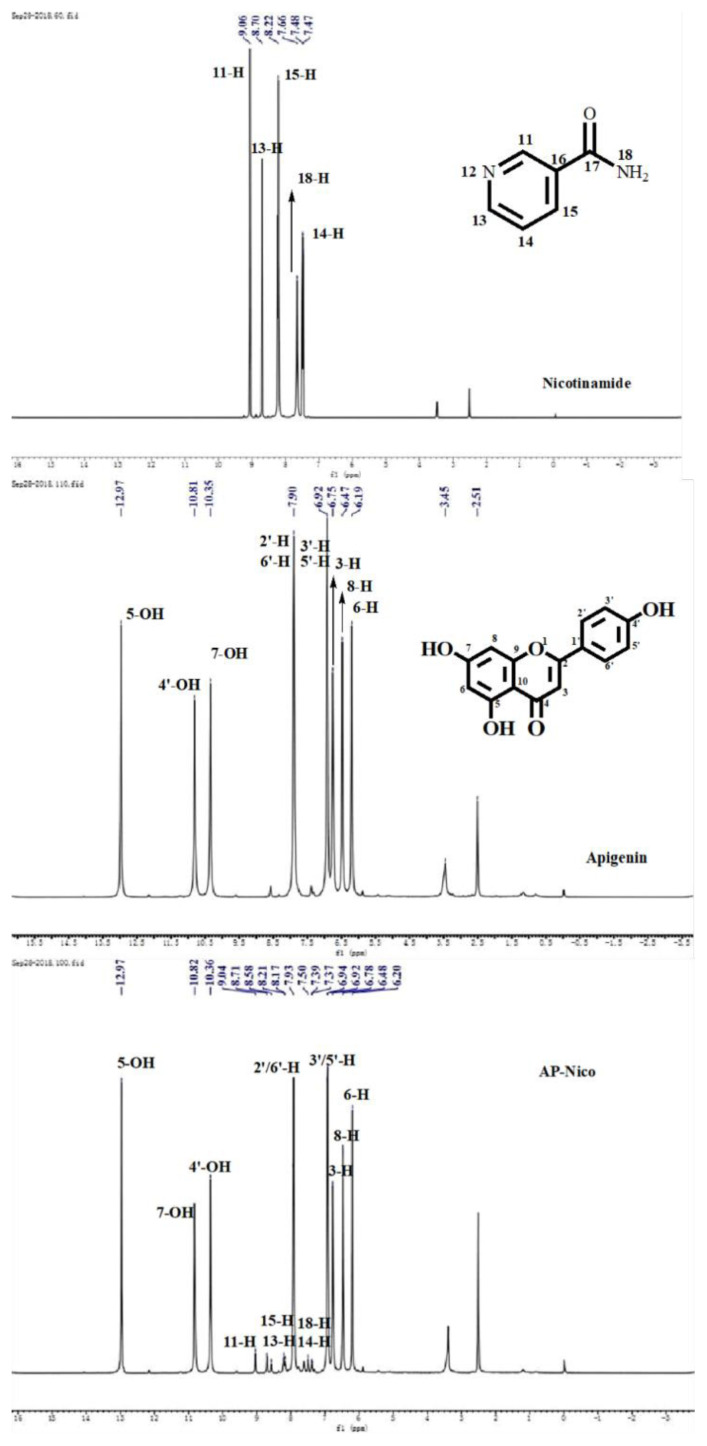
^1^H-NMR spectra of Nico, AP, and AP-Nico.

As shown in Figure 3, ^1^H-NMR (DMSO-d_6_, 500 MHz): Apigenin: 12.97 (1 H, s, 5 -H), 10.81 (1 H, s, 7 -H), 10.35 (1 H, s, 4′-H), 7.91 (2 H, d, 2′-H, 6′-H), 6.93 (1 H, s, 3′-H, 5′-H), 6.77 (1 H, s, 3 -H), 6.48 (1 H, s, 8 -H), 6.19 (1 H, s, 6 -H). Nico: 9.06 (1 H, s, 2 -H), 8.78 (1 H, s, 6 -H), 8.24 (1 H, s, 4 -H), 8.22 (1 H, s, 8 -H), 7.77 (1 H, s, 8 -H), 7.52 (1 H, s, 5 -H). AP-Nico: 12.76 (1 H, s, 5 -H), 10.82 (1 H, s, 7 -H), 10.36 (1 H, s, 4′-H), 7.91(2 H, d, 2′-H, 6′-H), 6.94 (1 H, s, 3′-H, 5′-H), 6.78 (1 H, s, 3 -H), 6.48 (1 H, s, 8 -H), 6.20 (1 H, s, 6 -H); 9.04 (1 H, s, 2 -H), 8.70 (1 H, s, 6 -H), 8.21 (1 H, s, 4 -H), 8.19 (1 H, s, 8 -H), 7.70 (1 H, s, 8 -H), 7.63 (1 H, s, 5 -H) [32, 33].

The ascription of NMR spectra is compared with the corresponding monomers. According to the results, the obtained product contained characteristic peaks of AP and Nico raw materials, indicating the presence of AP and Nico in the product, and the chemical shift of 5 -H (12.97) changed more obviously (Δδ = 0.21) than other groups on the AP ring, to Nico, the pyridine ring is more obvious than amide group on the Nico. In the formed process of pharmaceutical cocrystal, there is no chemical bond formed between molecules and there are only weaker intermolecular forces, including dipole-dipole, electrostatic and hydrogen bonding interaction force; therefore, the chemical shift is not obvious (< 1 ppm) when the pharmaceutical cocrystal formed. The chemical shifts of H atoms move to high fields [34], which shows that the electron cloud density of surrounding environment increases, there may be covalent binding, so it can be inferred that the site of interaction may exist interaction between 5 -H of AP and 5 -H of Nico.

According to the above product results, after Nico was introduced, the structural information of the products changed due to the covalent interaction between AP and Nico; thus, AP did interact with Nico. Presumably, the 5 -OH of AP was obviously covalently bound to -NH_2_ of Nico, and there was some interaction force with 5 -H of Nico. It is consistent with our purpose of experiments, so the physicochemical properties of the two compounds were compared by further solubility experiments.

#### 3.1.3. ^13^C-NMR characterization

As shown in Figure S2, ^13^C-NMR (DMSO-d_6_, 126 MHz): Apigenin: δ 181.77 (C-10), 164.15 (C-2,8), 161.51(C-6), 161.19 (C-14), 157.34 (C-4), 128.46 (C-12,16), 121.24 (C-11), 115.98 (C-13,15), 102.87 (C-5,9), 98.87 (C-1), 93.99 (C-3). Nico: δ 166.61 (C-22), 151.95 (C-21), 148.76 (C-17), 135.23 (C-19), 129.73 (C-20), 123.46 (C-18). AP-Nico: δ 181.86 (C-10), 166.81 (C-22), 164.25 (C-2,8),161.64 (C-6), 161.29 (C-14), 157.45 (C-4), 152.00 (C-21), 148.86 (C-17), 135.35 (C-19), 129.94 (C-20), 128.53 (C-12,16), 123.53 (C-18), 121.38 (C-11), 116.08 (C-13,15), 102.98 (C-5,9), 98.99 (C-1), 94.10 (C-3).

The ascription of ^13^C-NMR spectra is compared with the corresponding monomers. According to the results, the AP-Nico contained characteristic peaks of AP and Nico raw materials, indicating the presence of AP and Nico in the product. The chemical shifts of ^13^C-NMR were more sensitive to the stereochemical information response. In this study, the chemical shift of the carbonyl carbon (C-10) in AP shifted from 181.77 ppm to 181.86 ppm after cocrystal formation. The chemical shift of the carbonyl carbon linked to the N-H in Nico shifted from 166.61 ppm to 166.81 ppm after cocrystal formation. It indicated that the characteristic peaks of carbonyl carbon were shifted to the lower field during the formation of eutectic. The charge density on the carbonyl carbon atom is reduced by hydrogen bonding forces during AP-Nico pharmaceutical cocrystal formation [35]. The other carbon atoms also undergo slight chemical shifts in the formed process of pharmaceutical cocrystal, which indicated that molecular rearrangement had occurred and the chemical environment around the carbon atom had changed. The formation of pharmaceutical cocrystal improved the physicochemical properties of AP without breaking the original covalent bonds. Combined with the ^1^H-NMR results, it further confirms the production of AP-Nico pharmaceutical cocrystal.

#### 3.1.4. Elemental analysis

AP-Nico was prepared according to the ratio of n (AP): n (Nico)=1:1. The content of elements (C, H, and N) in organic matter was analyzed by the elemental analyzer. The calculated theoretical values were compared with the actual measured values, and the results are shown in Table S1. The measured values were basically consistent with the theoretical values, and the relative errors were within 5%.

#### 3.1.5. X-ray characterization

**Figure 4:**
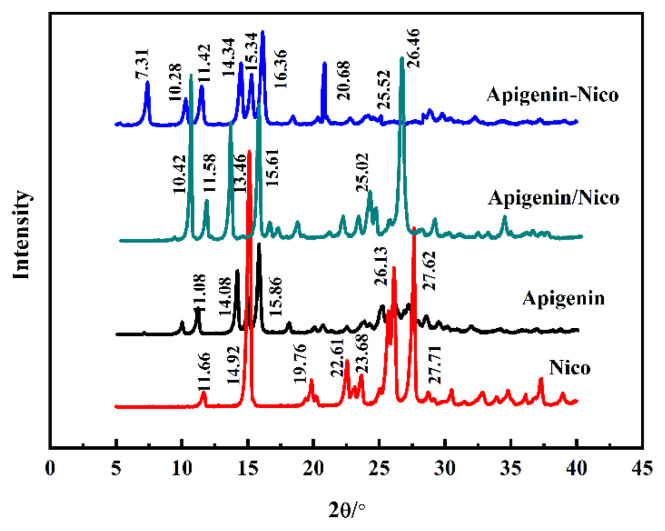
PXRD spectra of AP, Nico, AP/Nico, and AP-Nico.

PXRD analysis: The AP-Nico eutectic obtained after the conditional exploration was first characterized by PXRD (Figure 4), and it was again judged whether a new phase was formed.

In PXRD, a new set of diffraction peaks (compared with the starting material) indicate the formation of new crystalline phases. As shown in Figure 4, the PXRD patterns of the AP, Nico, the physical mixture of Nico and AP and AP-Nico pharmaceutical cocrystal. The characteristic peaks of AP are 11.08°, 14.08°, and 15.86°, respectively, and the characteristic peaks of Nico are 14.92°, 25.84°, and 27.71°, respectively. The obtained AP-Nico product is different from the physical mixture of the two, not a simple superposition of the characteristic peaks of the two, and the AP-Nico product has a new peak at 7.31°, 10.36°, 20.68°, indicating that the formed product has a new crystalline phase.

### 3.2. Crystalline state

DSC analysis: The possibility of forming pharmaceutical cocrystal could be elucidated by the thermal analysis in DSC. The purpose of DSC experiments was to study the thermal behavior of AP-Nico pharmaceutical cocrystal to screen the new forming pharmaceutical cocrystal.

**Figure 5:**
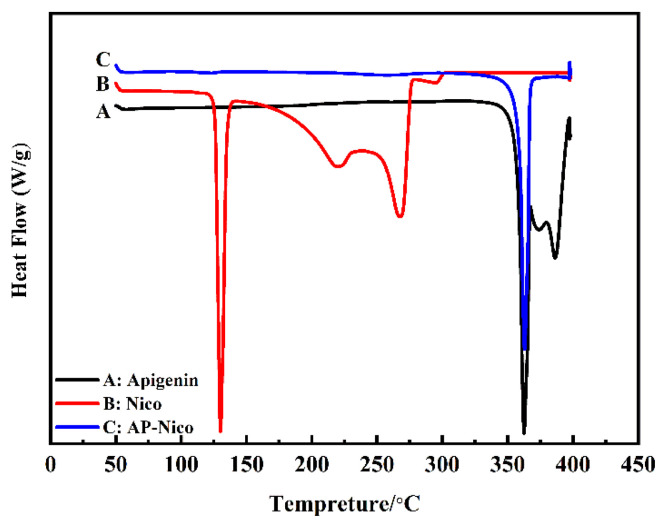
DSC profiles of apigenin, nicotinamide, and AP-Nico pharmaceutical cocrystal.

The DSC traces for the samples are presented in Figure 5. The results showed that AP had a characteristic peak at 362 K, which was the melting point. Nico had melting peaks at 129, 219, and 267 K, which is in line with the literature [36]. The AP-Nico pharmaceutical cocrystal prepared by the ethanol suspension showed a new melting peak at 298, 363, 374, and 386 K. Generally, the formation of AP-Nico indicates that the orderliness of crystal structure transforms into eutectic disorder, which leads to the disappearance of melting point and the appearance of amorphous phase.

Above, the results of DSC and X-ray showed that the amorphous state was formed during homogenization. Amorphous state drugs have higher solubility and faster dissolution rate due to the higher internal energy. Therefore, when the drug is administered orally, if it can maintain a high energy state, its effect on improving bioavailability will be more significant than that of the initial crystalline [37, 38].

### 3.3. Dissolution test of the AP-Nico pharmaceutical cocrystal in vitro

The drug nanocrystals are a noncovalent derivative that enhances the physical properties of an active pharmaceutical ingredient. Without changing the molecular structure of the drug, it can not only retain the drug properties of the cocrystal but also improve the physicochemical properties of the drug and improve the clinical efficacy [39]. The poor solubility of API is the main reason for its low bioavailability in vivo. An important characteristic of drug nanocrystals is the increase of saturated solubility and dissolution rate [40], which makes drug nanocrystals suitable for many applications. The solubility of the Ap-Nico system is optimized by cocrystallization, which promotes the rapid absorption of drug molecules in the system circulation, and is better absorbed by the organism [41], thereby increasing the relative bioavailability of the drug. Thus, the dissolution experiment was carried out to estimate the dissolution profiles of AP-Nico pharmaceutical cocrystal. The apparent solubility of crystalline AP and AP-Nico pharmaceutical cocrystal after 72 h in a 50:50 ethanol-water mixture (*v*/*v*) were assessed at different temperatures (Table 1). AP-Nico pharmaceutical cocrystal was more soluble than pure AP throughout comparison of solubility curves which defined standard concentration of AP determined in a solution. As shown in Table 1, AP was almost insoluble in water (0.23–0.28 × 10^−5^ mol/L); however, the solubility of AP-Nico pharmaceutical cocrystal showed a significant increase about 0.57–0.81 × 10^−5^ mol/L. In addition, when the temperature rose from 298 K to 310 K, the solubility of AP was almost invariable while the solubility of AP-Nico had a relatively obvious increase. As a whole, the apparent solubility of AP-Nico pharmaceutical cocrystal was about 3 times higher than that of crystalline AP. The possible reason was that there may be different interaction forces between AP-Nico supermolecular systems due to the introduced CCF-Nico in the process of suspension, the AP and Nico were arranged by the interaction forces, and the AP-Nico pharmaceutical cocrystalline phase exhibited thermal stability AP-Nico.

**Figure 6:**
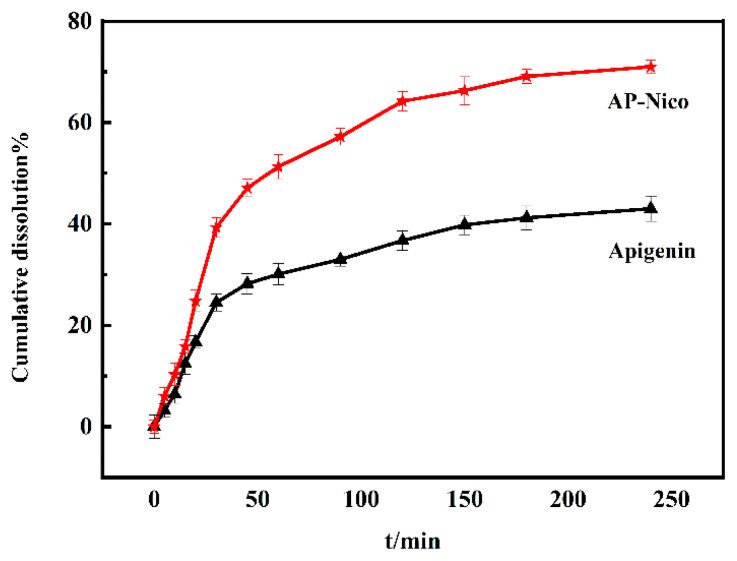
Dissolution profiles of AP/AP-Nico in vivo (pH 4.5).

Cumulative dissolution: The dissolution behaviors of AP and AP-Nico under the physiological simulated conditions are shown in Figure 6. The results showed that the accumulative dissolution rate of AP was 43% at pH 4.5 after 4 h, but it could even reach 71% for AP-Nico pharmaceutical cocrystal. On the other hand, the dissolution speed of AP-Nico was faster than AP before 2 h. The phenomenon showed that the cumulative dissolution of AP-Nico pharmaceutical cocrystal was higher than that of crystalline AP [42]. As mentioned above, when the -OH group of AP combined with the -NH_2_ group of Nico, it formed noncovalent bonds in -H_2_N…H-O- in the suspension. The AP and Nico assembled into a network structure with certain dimensions, resulting in the lattic stacking and molecular arrangement of the original AP had been changed. Thus, the AP-Nico pharmaceutical cocrystal can reduce the bioavailability of individual difference to so.

Therefore, nicotinamide, 2-aminometidine, and citric acid are commonly used to screen for the biocompatibility of eutectic CCF, which contain - COOH, - OH, - NH_2_, and other active groups. Apigenin can form eutectic compounds by introducing CCF through intermolecular hydrogen bonds. Based on the above theory, the apigenin-nicotinamide eutectic was successfully screened out. PXRD results screened out compounds with new crystalline phase; FTIR analysis showed that the weak hydrogen bonding force in the complex was mainly due to the covalent interaction between - OH in apigenin and - NH_2_ or - C = O in CF; DSC results showed new endothermic peaks; PXRD, FTIR, NMR, and DSC characterization results confirmed the successful synthesis of the expected products. The solubility test showed that the solubility of AP-Nico was 3 times higher than that of raw AP, and the cumulative release rate in vitro was 71%. The dissolution rate of insoluble active substance group is superior to that of raw materials. It is speculated that the mechanism may be due to the preferential release of soluble CCF when API and CCF form eutectic through noncovalent hydrogen bond interaction, which destroys the lattice formed by them, while the insoluble API is in an amorphous state (disorder, random orientation), which increases the entropy value and bonding of API molecules. The contact area accelerated the dissolution rate of API. Successful screening of insoluble active substances will lay a foundation for the further study of intermolecular interaction with biological macromolecules.

### 3.4. Drug delivery of AP-Nico pharmaceutical cocrystal

The persistency and bioaccumulative properties of drugs result in easy absorption through accumulation in blood, potentially causing interactions with plasma proteins. The transport, distribution, and metabolism of many exogenous ligands are highly dependent on their binding to the most abundant transporter HSA in human plasma. As the most abundant protein in plasma, HSA plays an important role in the transport and storage of endogenous metabolites and exogenous drug molecules [43].

### 3.5. Fluorescence emission data analysis

Fluorescence spectroscopy method is an excellent tool to investigate the binding constant, binding sites and binding mechanism in proteins upon association with AP-Nico pharmaceutical cocrystal [44, 45]. This method can reveal the quenching effect of fluorophores on proteins, understand the binding mode of proteins and compounds, and provide clues for the properties of their binding mechanism. In the HSA, tryptophan residue (Trp 214) s projected lie in the hydrophobic pocket of subdomain IIA (Sudlow’s binding site I), which contributes to intrinsic fluorescence.

**Figure 7:**
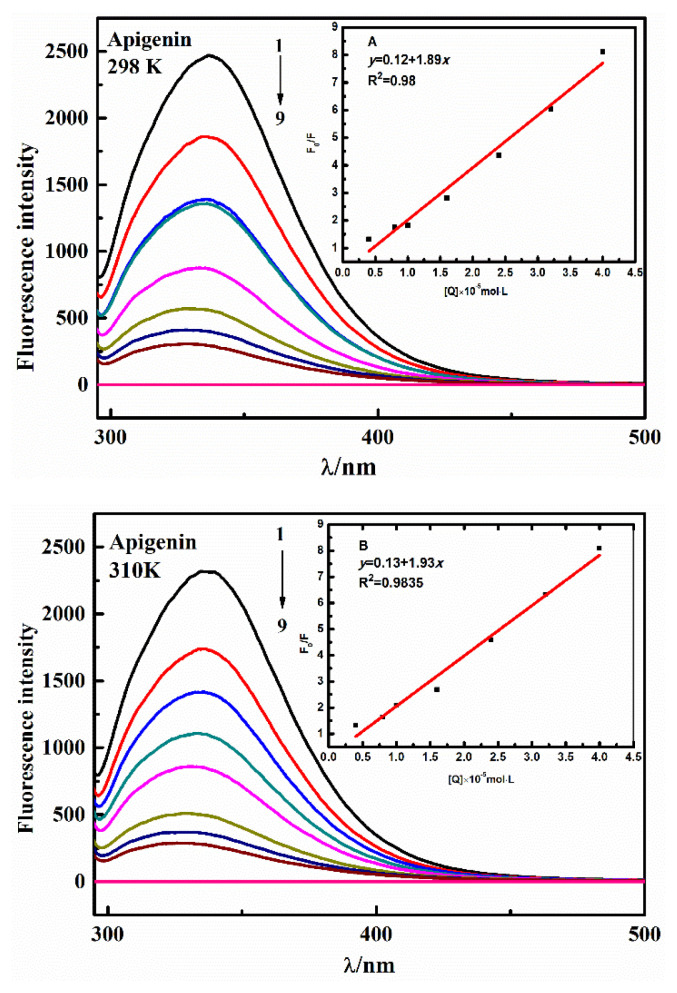
The fluorescence emission spectrum of AP-HSA complex was measured at physiological pH 7.4. Conditions: λex = 282 nm, cHSA = 1.0 × 10^−5^ mol L^−1^, cAP (×10^−5^ mol L^−1^), 1 to 8: 0, 0.40, 0.80, 1.00, 1.60, 2.40, 3.2, 4.00. The line of 9 is the fluorescence of AP. [The up-right diagram is the Stern-Volmer fitting plot of AP-HSA system.]

**Figure 8:**
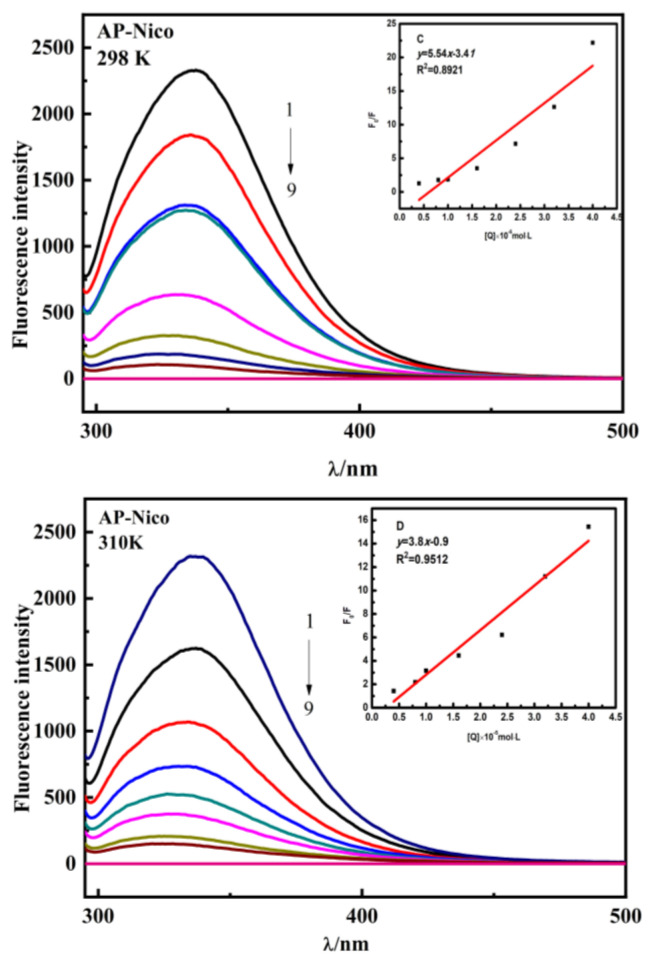
The fluorescence emission spectrum of AP-Nico-HSA complex was measured at physiological pH 7.4. Conditions: λ_ex_ = 282 *nm*, *c*_HSA_ = 1.0 × 10^−5^mol L^−1^, *c*_AP_ (×10^−5^mol L^−1^), 1 to 8: 0, 0.40, 0.80, 1.00, 1.60, 2.40, 3.2, 4.00. The line of 9 is the fluorescence of AP. [The up-right diagram is the Stern-Volmer fitting plot of AP-Nico-HSA system.

As shown in Figures 7 and 8, the fluorescence intensity of HSA is quenched with the increase of AP-Nico eutectic concentration. It is particularly noted that AP-Nico pharmaceutical cocrystal does not have fluorescence absorbance near the maximum emission wavelength of HSA. At the highest AP-Nico concentration, the fluorescence intensity changed and a slight blue shift occurred at the emission peak. The shift of emission maximum position corresponds to the change of polarity around chromophore molecules [46], which indicate that there existed a binding behavior between AP-Nico pharmaceutical cocrystal and HSA. Although the quenching fitting diagram of AP-Nico was also linear, the deviation was much larger than the correlation coefficient of the monomer AP, and the reason may be that AP and Nico formed AP-Nico supramolecular by intermolecular forces.

Meanwhile, different kinds of reactions such as energy transfer, excited state reaction, molecular rearrangement, complex stability, and collision quenching occur in the molecular process. It will be responsible for the quenching of HSA during drug binding with proteins. According to the Stern-Volmer equation [44], the quenching mechanism of AP-Nico and HSA interaction could be explored.


(3) $$F_{0}/F=1+K_{sv}[Q]=1+K_{q}\tau_{0}[Q],$$

where *F*_0_ and *F* stand for the fluorescence intensity in the absence and presence of ligand, respectively; *K_SV_* (mol/L) is Stern-Volmer constant; *K_q_* [L / (mol·s)] is the quenching rate constant of biomacromolecule; τ0 (s) is the total lifetime (1 × 10^−8^ s); Q (mol/L) is the concentration of AP/AP-Nico.

As shown in Table 2, the value of *K_q_* was significantly greater than the value of 2 × 10^10^ L/mol/s, indicating that the interaction of AP-Nico and HSA system was static quenching due to the formation of a stable complex. Moreover, the *K_SV_* value obviously was larger than AP, indicating that the interaction between AP-Nico pharmaceutical cocrystal was affected by the intermolecular interaction of AP and Nico, and about the further interaction mechanism, the molecular docking and MD simulation will explore as follows.

For static quenching, the AP-Nico pharmaceutical cocrystal binding affinity against the HSA was explored. The binding sites of different ligands have higher or lower affinity, and have more weak or medium affinity [25]. Generally, the range of good binding affinity was 10^5^ to 10^6^ L/mol. As shown in Table 3, the calculated *K* value of the AP-Nico pharmaceutical cocrystal was 4.57 × 10^6^ L/mol, there was a strong binding affinity between AP-Nico pharmaceutical cocrystal and HSA. This type of binding affinity facilitates the effective delivery of drugs and their subsequent release at target sites [44]. Besides, the *K* value of the AP-Nico-HSA system showed an increasing trend (a large an order of magnitude) which compared with AP-HSA system. It was contributed to the introduced of CCF-Nico, and the interaction between AP and Nico was affected by the interaction of AP-Nico with HSA. Sudlow et al. [47] first proposed that there were two major drug binding sites distributed on HSA, Sudlow’s site I and Sudlow’s site II. The efficacy of the drug in an organism was closely related to these two binding sites on the HSA. The active regions of HSA, Sudlow’s Site I and Sudlow’s Site II, were the main regions where the small molecules act, with warfarin bound to Sudlow’s Site I and ibuprofen bound to Sudlow’s Site II [45]. According to the results of binding site competition experiments (Table 4), the *K* value of AP-Nico-HSA changed with the addition of warfarin, which indicated that AP-Nico mainly binds to Sudlow’s site I of HSA.

### 3.6. Thermodynamic parameters

The binding parameters of AP-Nico system were further evaluated. The noncovalent bond could form a strong directional force under certain conditions, which was the basis of the combination of biomacromolecules and ligand [26]. The binding force between AP-Ni pharmaceutical cocrystal and HSA can be characterized by calculating thermodynamic parameters such as enthalpy change (Δ*H*), entropy changes (Δ*S*), and free energy (Δ*G*), which could explain the interaction for the system. Therefore, according to the Van’t Hoff thermodynamic formula, the thermodynamic parameters of AP-Nico-HSA systems can be obtained:


(4) ΔG=ΔH-T·ΔS,

(5) ΔG=-RT·lnK,

(6) $$\text{ln}(K_{2}/K_{1})=(\Delta H/R)\cdot (1/T_{1}-1/T_{2}),$$

where K (L/mol) represents binding constant; R is a gas constant of 8.314 J/mol/K. The interaction mechanism of AP-Nico-HSA system could be determined by the following rules: Δ*S* > 0 expressed as the existence of hydrophobic and electrostatic force, Δ*S* < 0 indicated the presence of hydrogen bonding and van der Waals force; Δ*H* > 0, Δ*S* > 0 suggested hydrophobic force; Δ*H* < 0, Δ*S* < 0 expressed as both hydrogen bonding and van der Waals force [44]. The negative value of G at different temperatures can clearly explain the complex formation of AP-Nico-HSA. The exothermic reaction binding of AP-Nico-HSA could be also characterized from the negative sign of Δ*H* values.

As shown in Table 5, the negative Δ*S* (−21.36 J/mol/K) may lead to greater disorder of solvent molecules surrounding the AP-Nico and HSA. The AP-Nico-HSA forces could be easily predicted from the sign and magnitude of the thermodynamic parameters [43, 44]. In broad perspective, the more negative value of Δ*H* (−14.45 J/mol/K) could associate with both hydrogen bonds and van der Waals forces contribute to the stabilization of AP-Nico-HSA complex. Earlier studies [48] had shown that undissociated small molecules were weakly bound to protein. The greater the degree of ionization of the anion, the greater the electrostatic attraction towards the cation center in the protein. van der Waals forces had a greater effect on the binding of these small molecules than electrostatic effects. In supramolecular systems, the binding between AP and Nico was through noncovalent weak forces such as hydrogen bonding and van der Waals forces. The combination of AP-Nico and HSA was also a supramolecular system, and the two were also bound together based on weak forces such as hydrogen bonding, van der Waals forces, and hydrophobic interactions. The weak interaction between AP and Nico affected its binding to HSA. Among them, van der Waals force was an important factor in enhancing the binding effect of AP-Nico to HSA. Therefore, the hydrogen bonds and hydrophobic interactions are mainly responsible for the complex of the AP-Nico-HSA. Similarly, the result is also supported by the molecular docking and MD simulation.

### 3.7. Molecular docking studies

**Figure 10:**
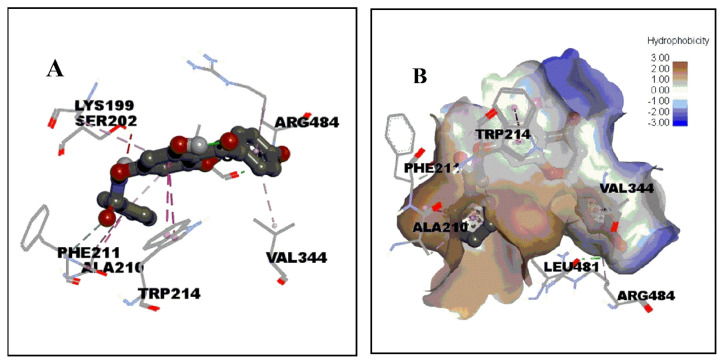
The optimal energy score of molecule docking results for AP-Nico-HSA system (A); the hydrophobic surface map of AP-Nico-HSA system (B).

To further explore the molecular interaction mechanism of ligands to HSA at atomic level through molecular docking, it allows the prediction of the binding pattern of AP-Nico pharmaceutical cocrystal in the binding site of HSA. The molecular docking is shown in ure 9 and Figure 10.

The molecular docking analyses for AP-Nico and AP-HAS are shown in Table 6. Anchored AP-Nico to Sudlow’s Site I pocket composed of 18 amino acids residues within 4 Å, including Phe 206, Phe 211, Lys 199, Trp 214, Lys 195, Ala 210, Ser 202, Leu 481, Val 482, Pro 486, Arg 348, Arg 485, Val 344, Arg 484, Glu 450, Ser 454, Leu 198, Asp 451. It was especially noted that Phe 206, Phe 211, Trp 214, Ala 210, Leu 481, Val 482, Val 344, and Leu 198 residues formed a hydrophobic cavity. Furthermore, in the AP-Nico-HSA system, Ala 210 and Trp 214 residues conjugated with benezene ring of Nico and A ring of AP to form π-π bond, respectively (2.1 Å, 4.1 Å), and the Val344 and Lys199 residues interacted with the AP’s rings of B and A with distances of 4.3 and 5.4 Å, respectively, which formed amide-π force. Meanwhile, van der Waals also would be a major factor which stabilizes the complex of AP-Nico-HSA by Phe206, Phe211, Lys195, Val482, Pro486, Arg348, Arg485, Glu450, Ser454, Leu198, Asp451 moieties [45]. Compared to the initial AP interaction with HAS, the docking results were different, indicating that Nico introduced was affected by the interaction between AP and HSA. And this was in line with the above experiments. Besides, compared with the theoretical and experimental values of ΔG, there was not much deviation between them, which showed that the molecular modeling of AP-Nico-HSA model was reasonable.

### 3.8. MD simulation analysis

**Figure 11:**
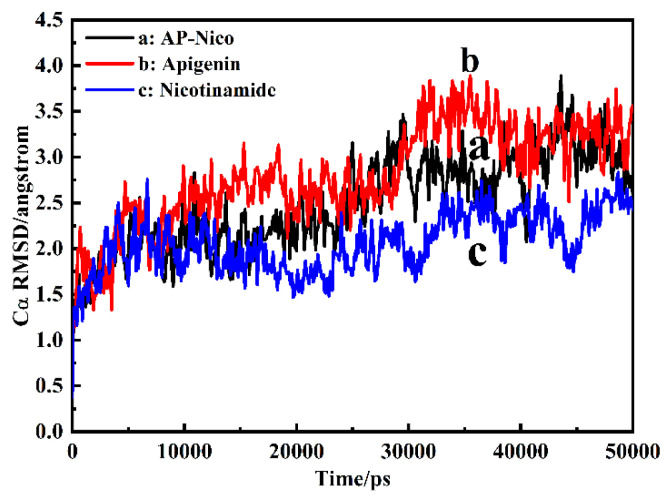
The C_α_ RMSD monitored over 50 ns MD simulations trajectories (a: AP-Nico, b: Apigenin, c: Nico). l cocrystalo)

Molecular dynamics (MD) simulation is the best method for stability analysis of biomolecular docking complexes [49]. To further study the conformational spaces of HSA interacted with AP-Nico pharmaceutical cocrystal, and verify the model of theoretical system using MD simulation in vitro. The apo HSA and AP-Nico bound HSA to quantitatively probe the local conformations of biomolecules through obtaining the root mean square deviation (RMSD) of C_α_ atoms of residues and the root mean square fluctuation (RMSF) of amino-acid residues [25]. MD simulations of HSA with Ap-Nico pharmaceutical cocrystal were performed in 50 ns. The conformational changes of HSA in AP-Nico pharmaceutical cocrystal are depicted in Figure 11.

As shown in Figure 11, the average C_α_ RMSD of apo HSA is 2.06 Å, indicating a relatively rigid and stable feature, following bound with AP and AP-Nico, the average C_α_ RMSD changed to 2.93 Å and 2.53 Å, the slight change C_α_ RMSD indicating that the interaction between AP-Nico and HSA, and the RMSD value of AP-Nico eutectic system is the lowest than that of APO-HSA and RAW-AP, which further confirms the authenticity of the docking results. Besides, the RMSD of the AP-Nico system achieved equilibrium at 30 ns, and there was no more evident fluctuation afterwards process than AP-Nico-HSA system, it also indicates the AP-Nico ligand associated with HSA binding at surface sites [50], which is consistent with the above mentioned fluorescence spectroscopy.

## 4. Conclusion

The paper provided a further insight into crystal engineering of AP by obtaining and characterizing its pharmaceutical cocrystal with Nico. The results revealed that the hydrogen bonds between -OH group of AP and −NH_2_ group of Nico in AP-Nico pharmaceutical cocrystal with a mole ratio of 1:1. The solubility of the pharmaceutical cocrystals was 3 times higher than the raw AP, and the cumulative release rate of AP-Nico pharmaceutical cocrystals reached 71% in vitro. The synthetic AP-Nico also showed a stable thermal stability. In vitro and in silico studies both showed that AP-Nico bonded to HSA tightly at Sudlow’s site I. AP-Nico pharmaceutical cocrystals could evidently quench the intrinsic fluorescence of HSA by a static quenching mechanism. The fluorescence spectra result indicated the interaction between AP-Nico pharmaceutical cocrystal and HSA, resulting in specific amino acid residues. The AP-Nico pharmaceutical cocrystal bound at Sudlow’s site I consequently forming π-π conjugation, which improved the binding affinities. Meanwhile, the hydrophobic interactions are the main force, whereas van der Waals and hydrogen bonds play a key role for the interaction between AP-Nico pharmaceutical cocrystal and HSA. Therefore, AP-Nico pharmaceutical cocrystal is firstly reported compared to other natural flavonoid. The above work is of great significance for providing beneficial information to improve the solubility and bioavailability of natural drugs and evaluate the pharmacological mechanisms of drugs.

## Supplementary Information

**Table S1:** Elemental analysis results of AP, Nico and AP-Nico

Element		Sample		
		AP	Nico	AP-Nico
*w*(C)%	Theoretical	66.67	59.02	64.28
	Measured	64.68	58.50	64.47
*w*(H)%	Theoretical	3.70	4.92	4.11
	Measured	3.85	5.02	3.98
*w*(N)%	Theoretical	/	22.95	7.14
	Measured	/	22.94	6.93

Figure S1 The reaction diagram of AP-Nico pharmaceutical-cocrystal

Figure S2 13C-NMR spectra of AP, Nico and AP-Nico

## Figures and Tables

**Figure 9:**
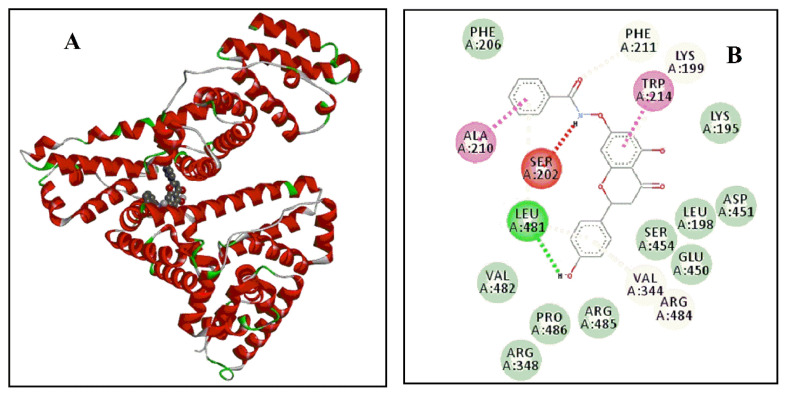
The binding domain diagram of AP-Nico-HSA system using the molecular docking method (A); the binding pocket of AP-Nico-HSA system within 5Å distance (B).

**Table 1:** Solubilities of apgenin and AP-Nico.

*T*/(K)	Apgenine(1 × 10^−5^mol/L)	AP-Nico (1 × 10^−5^mol/L)
278	0.23	0.57
310	0.28	0.81

**Table 2:** Quenching parameters of AP-Nico/AP-HSA intermolecular interaction at different temperatures.

*T*/(K)	*K*_SV_/(10^5^ L mol^−1^)		*K*_q_/(10^13^ L mol^−1^ s^−1^)	
	AP	AP-Nico	AP	AP-Nico
298	0.27	5.54	0.27	5.54
310	0.13	3.82	0.13	3.82

**Table 3:** Binding constant *K*_b_ and the number of binding sites n of the AP and its complex-HSA system at different temperatures.

Samples	*T*/K	lg[(*F*_0_-*F*)/*F*]=lg*K*+nlg[*Q*]	*R* *^2^*	*K*_b_/(10^6^L mol^−1^)	*n*
AP	298	*y*=6.83+1.36*x*	0.9903	0.67	1.36
	310	*y*=7.38+1.46*x*	0.9826	2.39	1.46
AP-Nico	298	*y*=7.66+1.48*x*	0.9867	4.57	1.48
	310	*y*=9.741.93*x*	0.9809	5.01	1.93

**Table 4:** Binding constants of binding site competition experiment of AP-Nico/AP-has.

System	*T*/(K)	Site probe	*K*/( L mol^−1^)	*R* ^2^
AP	298	Warfarin	0.57×10^5^	0.9912
AP	298	Ibuprofen	1.89×10^5^	0.9823
AP-Nico	298	Warfarin	1.05×10^5^	0.9951
AP-Nico	298	Ibuprofen	1.73×10^5^	0.9993

**Table 5:** Thermodynamic parameters of AP-Nico/AP-HSA intermolecular interaction at different temperatures.

System	*T*/(K)	Δ*H*/(kJ mol^−1^)	Δ*S*/(J mol^−1^·K^−1^)	Δ*G*/(kJ mol^−1^)
AP	298	−47.34	−59.91	−33.28
	310			−34.34
AP-Nico	298	−14.45	−21.36	−38.47
	310			−36.56

**Table 6:** Molecular docking analysis for AP-Nico/AP-HSA complexes system.

Interaction model	Binding pocket	Acceptor residues	Bonding involved	Δ*G*/(kJ/mol)	Δ*G′*/(kJ/mol)
AP-Nico-HSA	Sudlow’s Site I	Phe206, Phe211, Lys199, Trp214, Lys195, Ala210, Ser202, Leu481, Val482, Pro486, Arg348, Arg485, Val344, Arg484, Glu450, Ser454, Leu198, Asp451	van der Waals, hydrogen bond, π-π stacked, π-alkyl, amide-π stacked	−36.75	−38.47
AP-HAS	Sudlow’s Site I	Lye195, Leu198, Lys 199, Ser202, Ala210, Phe211, Leu203, Gly207, Ser454, Trp214, Val344, Leu481, Phe206	van der Waals, hydrogen bond, π-σ, π-alkyl	−32.94	−33.28

Note: ΔG: It represents the theoretical value of molecular modeling. ΔG′: It represents the actual value of experiments.
