# Supplementary figures and images for: Imaging supermolecular interactions of the pharmaceutical-cocrystal of apigenin-nicotinamide binding with serum albumin
Source: Turk J Chem. 2023 Apr 4;47(3):554–71. doi: 10.55730/1300-0527.3560 (PMC10387959; doi:10.55730/1300-0527.3560)

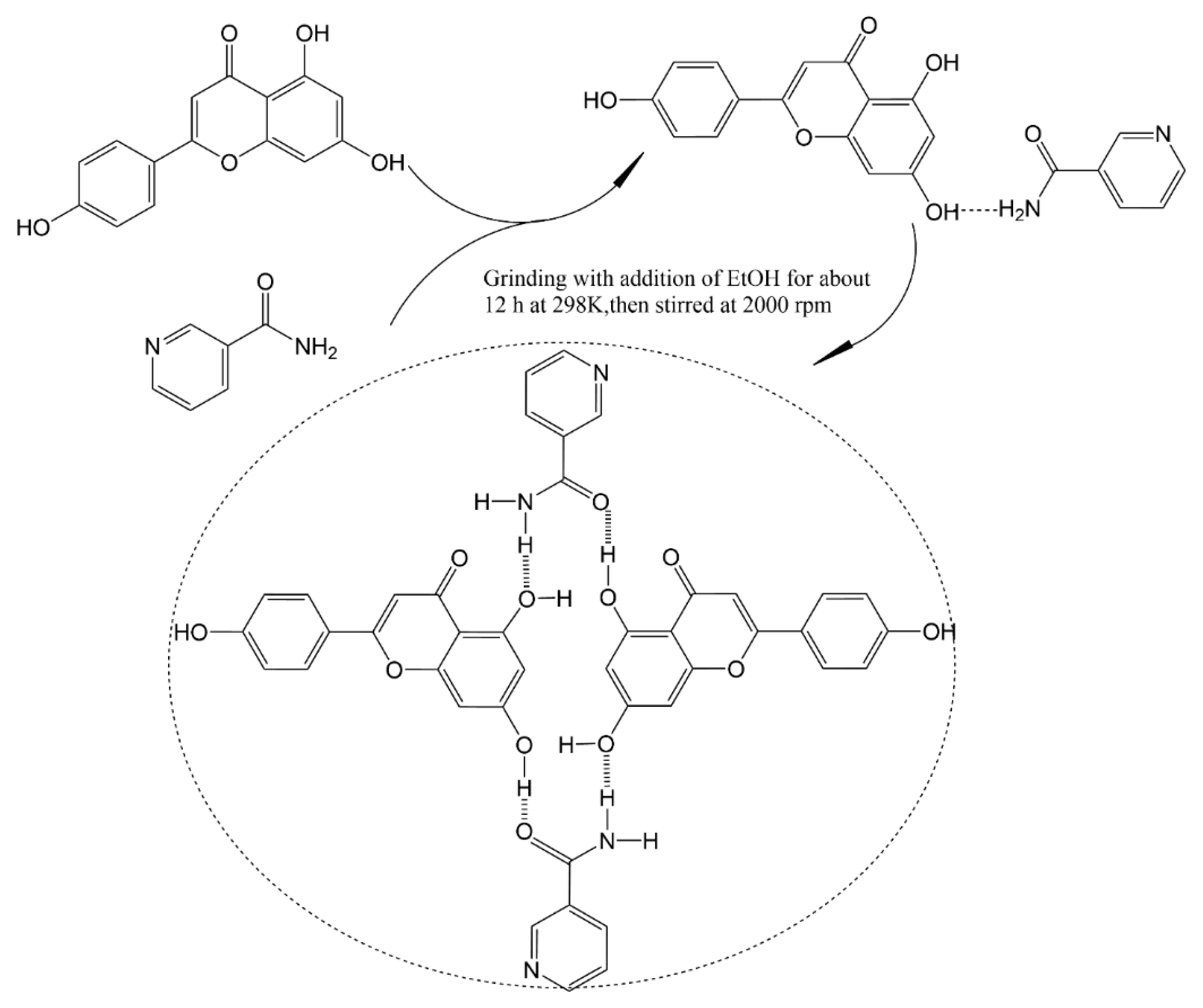

Supplement: Figure S1 — The reaction diagram of AP-Nico pharmaceutical-cocrystal [file turkjchem-47-3-554s1.tif]

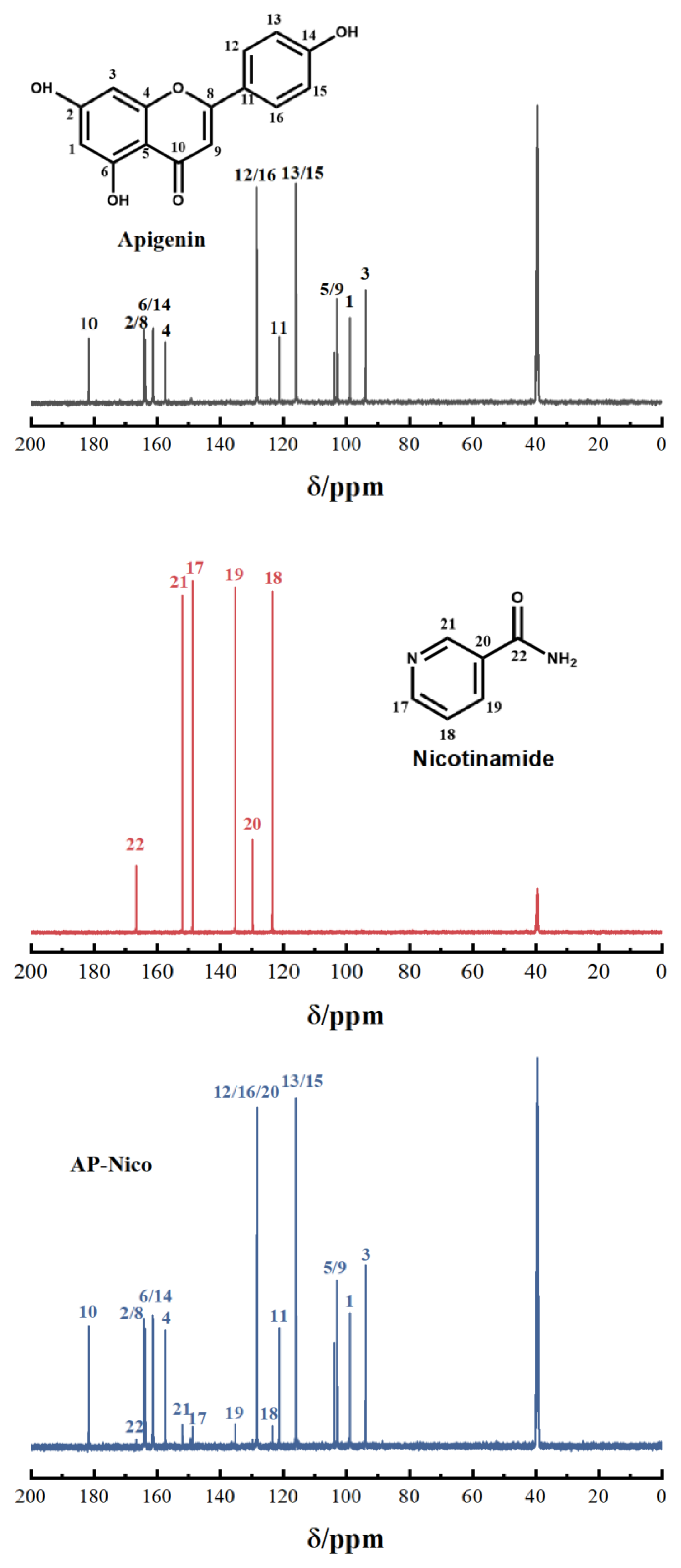

Supplement: Figure S2 — 13C-NMR spectra of AP, Nico and AP-Nico [file turkjchem-47-3-554s2.tif]
